# Fat embolism in right internal jugular vein: incidental ultrasound finding during internal jugular vein cannulation

**DOI:** 10.1186/s13089-019-0116-9

**Published:** 2019-02-25

**Authors:** Osman Adi, Chan Pei Fong, Azlizawati Azil, Shaik Farid Abdul Wahab

**Affiliations:** 1 Department of Trauma and Emergency Medicine, Raja Permaisuri Bainun Hospital, 30450 Ipoh, Perak Malaysia; 2 Departments of Emergency Medicine, School of Medical Sciences, University Science of Malaysia (USM), Kubang Kerian, Kelantan Malaysia

**Keywords:** Fat embolism syndrome, Fracture shaft of femur, Point-of-care ultrasound

## Abstract

**Background:**

We report a case study of fat embolism seen on ultrasound at right internal jugular vein during central venous cannulation in a patient diagnosed with fat embolism syndrome. This case demonstrates the importance of ultrasound for evaluation of trauma cases with suspicion of fat embolism.

**Case presentation:**

A 23-year-old trauma patient with closed fracture of left femoral shaft and left humerus presented to our emergency department (ED). 11 h after admission to ED, patient became confused, hypoxic and hypotensive. He was then intubated for respiratory failure and mechanically ventilated. Transesophageal ultrasound revealed hyperdynamic heart, dilated right ventricle with no regional wall abnormalities and no major aorta injuries. Whole-body computed tomography was normal. During central venous cannulation of right internal jugular vein (IJV), we found free floating mobile hyperechoic spots, located at the anterior part of the vein. A diagnosis of fat embolism syndrome later was made based on the clinical presentation of long bone fractures and fat globulin in the blood. Despite aggressive fluid resuscitation, patient was a non-responder and needed vasopressor infusion for persistent shock. Blood aspirated during cannulation from the IJV revealed a fat globule. Patient underwent uneventful orthopedic procedures and was discharged well on day 5 of admission.

**Conclusions:**

Point-of-care ultrasound findings of fat embolism in central vein can facilitate and increase the suspicion of fat embolism syndrome.

**Electronic supplementary material:**

The online version of this article (10.1186/s13089-019-0116-9) contains supplementary material, which is available to authorized users.

## Background

Fat embolism is a relatively under-recognized condition in patients with long bone fractures. When symptomatic, it can lead to serious effects on vital organs and even death. Diagnosis of fat embolism remains a challenge to clinicians today, as multiple diagnostic criteria exist. We report a case where the use of point-of-care ultrasound was able to visualize fat deposited at right internal jugular vein and increase the index of suspicion in diagnosis fat embolism syndrome. This case illustration demonstrates the importance of ultrasound as a tool to evaluate trauma cases with suspicion of fat embolism.

## Case presentation

**Fig. 1:**
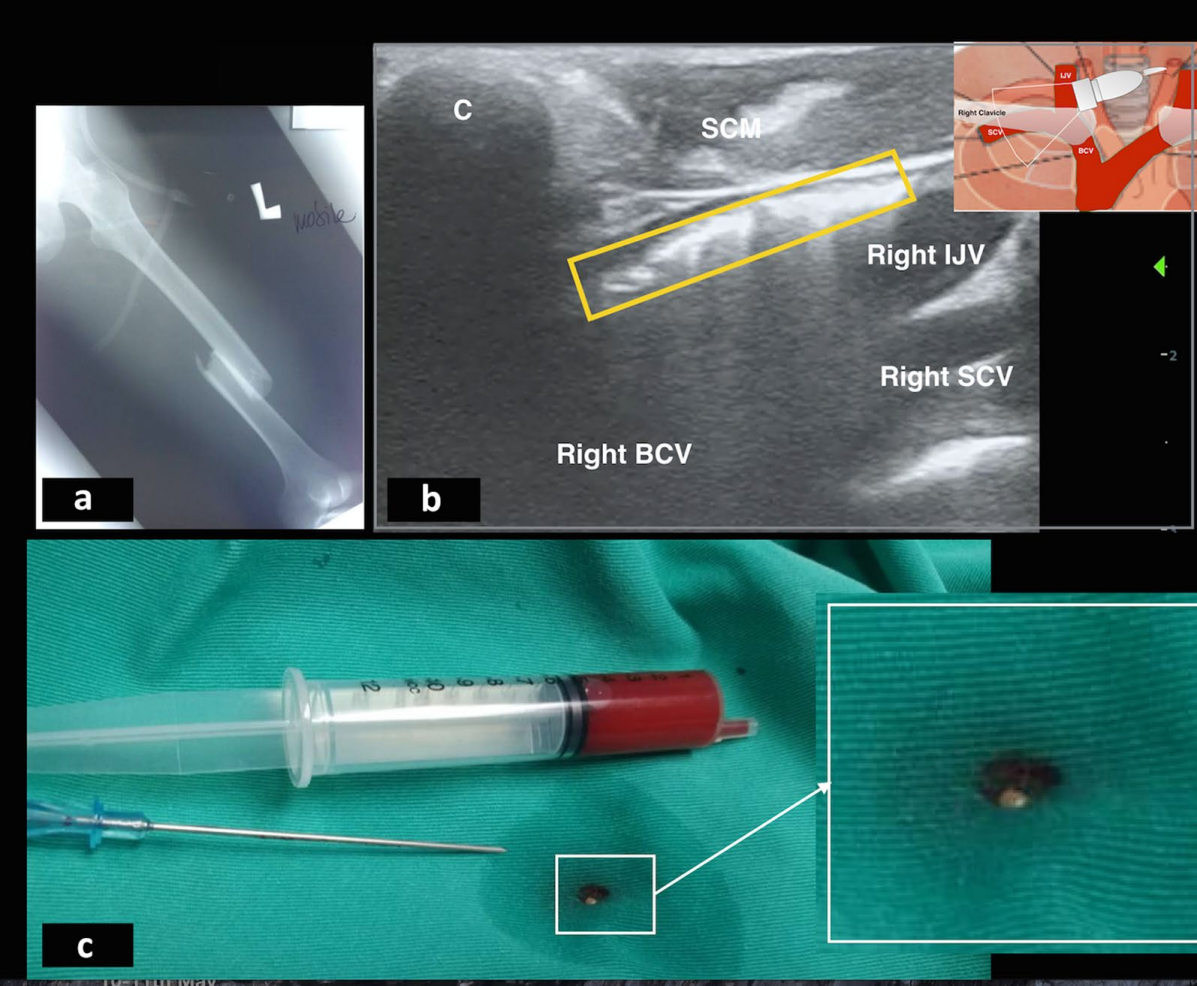
**a** X-ray showed closed fracture right femoral shaft. **b** Ultrasound images revealed free floating mobile hyperechoic spots, located in the anterior part of the vein and cleared from the vein (yellow box). Small picture showing probe location in relation to clavicle, internal jugular vein, subclavian vein and brachiocephalic vein. **c** Fat globule was aspirated during cannulation of the IJV as shown in the white box. *C* Clavicle, *IJV* internal jugular vein, *SCV* subclavian vein, *BCV* brachiocephalic vein

A 23 years old with no known medical illness presented to our emergency department after a head-on collision. He sustained closed fracture of the left femur (Fig. 1a), closed fracture of the left humerus, and multiple open fractures of the left foot. Patient was pink, not tachypneic and hemodynamically stable upon presentation. Patient was started on infusion tramadol for pain management and was planned for surgery.

11 h after ED admission, he was noted to be tachypneic and hypotensive with severe tachycardia. ABG showed hypoxemia and metabolic acidosis, despite O_2_ given via non-rebreathing mask. He was intubated and mechanically ventilated. Extended Focused Sonography for Trauma was normal. Bedside transesophageal ultrasound revealed hyperdynamic heart, dilated right ventricle with no regional wall abnormalities and no major aorta injuries. Computed tomography of the whole body was also normal.

During central venous cannulation of the right internal jugular vein (IJV), we found multiple mobile and floating hyperechoic shadows deposited at the anterior part of the right IJV (Fig. 1b, Additional file 1: Video S1) Blood aspirated during cannulation from the right IJV revealed a fat globule (Fig. 1c). A diagnosis of fat embolism was suspected based on these incidental ultrasound findings in relation to the clinical presentation and background of multiple long bone fractures. Despite aggressive fluid resuscitation, patient was a non-responder. He needed intravenous noradrenaline infusion for persistent hypotension and tachycardia.

In the intensive care unit (ICU), patient’s hemodynamic and respiratory status improved after continuous fluid and vasopressor therapy. Patient underwent multiple uneventful orthopedic procedures and was discharged well on day 5 of admission.

## Discussion

Fat embolism is defined as evidence of fat globules found in the lung parenchyma and blood circulation usually occurring in trauma patients with long bone fractures [1]. A small number of these patients may develop fat embolism syndrome (FES), a potentially life-threatening condition characterized by the classic triad of hypoxemia, neurologic abnormalities, and petechial rash [2]. The onset of symptoms generally occurs within 12–72 h of the trauma [3]. Currently, to our knowledge, there is no literature discussing the difference between thrombus and fat embolism. Thrombus tends to be larger, mobile, be somewhat less echo-dense, and more commonly associated with spontaneous echo contrast. In contrast, fat embolism ultrasound images revealed free floating small, hypermobile and hyperechoic spots. Earlier literature classified fat embolism into 3 grades with grade III being the more serious clinical FES that leads to respiratory failure compared to grades I and II which are harmless [4].

In a retrospective study from 1979 to 2005 at the United States, Stein et al. reported incidence of fat embolism syndrome (FES) to be 0.12% to 0.7% in patients with fractures. Multiple fractures are found to increase the risk of FES compared to isolated fractures with femur being the most common cause [5]. An earlier 10-year review in New York also showed that 0.9% of patients with long bone fractures had clinically apparent FES. However, the true numbers of FES are probably underestimated because of the difficulty in making a definite diagnosis and the lack of specific testing [1].

Diagnosis of fat embolism syndrome remains a challenge to clinicians today, as multiple diagnostic criteria exist. The most commonly used Gurd and Wilson’s criteria since 1974 proposed that 2 major criteria or 1 major and 4 minor criteria suggest a diagnosis of FES [6]. Major clinical features are (i) petechial rash (ii) respiratory symptoms plus bilateral signs on chest examination with positive radiographic changes, and (iii) cerebral signs unrelated to head injury or other condition. Respiratory findings were described as hypoxemia (PaO_2_ < 60 mmHg) on FiO2 of 0.4. Minor features are fever > 38.5, tachycardia HR > 110 beats/min, retinal changes (fat or petechiae), renal changes (anuria, oliguria, fat globules), sudden drop in hemoglobin, sudden thrombocytopenia, high erythrocyte sedimentation rate, jaundice and fat globules in the sputum.

The limitations of the existing diagnostic criteria are the lack of laboratory markers or imaging tests to confirm the diagnosis of FES. Thus, the diagnosis of fat embolism is still based on clinical discretion of the treating physician after excluding other causes [7, 8]. This case may open up new possibility of point-of-care ultrasound as a triage tool in the emergency department to raise the suspicion of patients with suspected FES. The ultrasound identification of fat embolism with or without fat aspiration will definitely inflate the possibility of FES. Ultrasound can be amalgamated with the existing clinical criteria to confirm the diagnosis of FES.

The improvement of the quality of transthoracic echocardiography nowadays makes it a useful tool to assess for fat emboli in the circulation and heart of patients with long bone fractures as demonstrated in multiple case reports [9–11]. Fat emboli had been described as mobile, echogenic intraluminal mass in vessels [9] and “snowstorm”-like appearance in the chambers of the heart [10]. Fat emboli are usually spherical in shape, non-obstructive and show phasic flow with breathing [11]. This can be distinguished from a thrombus which adheres to the vein wall leading to a non-compressible vein and non-phasic flow with breathing.

It should be borne in mind that similar hyperechogenic spheres may also appear on ultrasound with infusion of blood products or agitated saline [12]. Another possibility for the echogenic materials is rouleaux formation due to stagnant red blood cells which is usually visualized at the junction of the valves and can be cleared away with compression of the vessel [13]. The right internal jugular vein in this patient was far from the valve area, compressible and the hyperechoic structures did not disappear immediately even with repeated scanning. Intraluminal air or air bubbles are another differential diagnosis for fat emboli. However, studies revealed that intraluminal air are short-lived (appear only within 3–5 of cardiac cycles) and diffuse in the lung when traversing the pulmonary circulation [14].

Based on our experience in this case, we propose searching for fat globules at deep veins near fracture sites such as the femoral vein in lower limb fractures and the IJV in upper limb fractures. There is currently no algorithm for point-of-care ultrasound in trauma patients to look for fat emboli. We will like to suggest scanning any vein proximal to the fracture site, followed by inferior vena cava and both sides of IJV. To confirm the diagnosis of fat emboli, ultrasound can serve as a useful tool to aspirate the fat globules under direct visualization. Larger systematic studies are needed to test out this hypothesis and determine whether diagnosis of fat embolism can be established earlier. This new idea may lead to prevention of FES complication and decrease in mortality.

To date, there is no proven treatment of FES, once it manifests in a patient. Prevention and high index of suspicion in high-risk patients are, therefore, needed to detect the condition early. Several measures had been identified as prophylaxis to prevent FES, but the quality of evidence is not strong. Hydration had been postulated to decrease the risk of FES due to the dilutional effect on the fat emboli and stress-related mediators [15]. Another recommendation is the use of corticosteroid for its anti-inflammatory effect to prevent FES [16] but its use remains controversial.

Although early operative fixation had been found to reduce the incidence of FES, the increased intramedullary pressure during fixation of fractures ironically increased the risk of FES [17]. Embolic materials were observed to pass through the heart by transesophageal echocardiography during orthopedic fixation of femur fractures with larger-size emboli (more than 10 mm) being more associated with increased mortality [18]. The mortality rate for fat embolism syndrome is around 7–20% in the literature [1, 19].

## Conclusions

Point-of-care ultrasound findings of fat embolism in central vein can facilitate and increase the suspicion of fat embolism syndrome. Early diagnosis can help with prompt supportive treatment and improve outcome of this potentially fatal condition.

## Additional file

**Additional file 1.** Multiple mobile and floating hyperechoic shadows deposited at the anterior part of the right IJV.
